# Supplementary material for: Content-rich biological network constructed by mining PubMed abstracts
Source: BMC Bioinformatics. 2004 Oct 8;5:147. doi: 10.1186/1471-2105-5-147 (PMC528731; doi:10.1186/1471-2105-5-147)
Supplement: Additional File 2 — The original results of the above study (non-essential files are deleted to keep the file size under the limit set by BMC bioinformatics). [file 1471-2105-5-147-S2.bz2 › chilibotAdditionalFile2/dip40/396ID10775655E9950/html/EGFR_TGFA.html]

 


 **EGFR** and **TGFA** 
  
Found 1071 abstracts in PubMed, retrieved 40.  
 

 What does Google say? 
 PDF only 
| .edu only 

---

**Interactive relationship** (e.g. stimulation, inhibition, etc)

**Inhibitory relationship**- Our results demonstrate that E4T and  **TGFa**  provoke decreased Cbl recruitment,  **EGFR**  ubiquitination and  **EGFR**  degradation compared to EGF.  Ref: 12829707 J Biol Chem< MedlineTA>J Biol Chem,
- We conclude that the enhanced  **EGFR**  recycling that has been observed in HER 14 cells following  **TGFa**  or E4T stimulation correlates with decreased  **EGFR**  ubiquitination and  **EGFR**  degradation,.  Ref: 12829707 J Biol Chem< MedlineTA>J Biol Chem,
- The inhibition effect of cotransfecting of antisense TGF alpha  [ **TGFA** ]  and antisense  **EGFR**  was remarkable as compared with that of antisense TGF alpha  [ **TGFA** ]  alone.  Ref: 12569632 Zhongguo Yi Xue Ke Xue Yuan Xue Bao, 1999
- Co treating PC 3 cells with quercetin significantly attenuated EGF and TGF alpha  [ **TGFA** ]  induced growth and phosphorylation of ErbB  [ **EGFR** ]  2, ErbB  [ **EGFR** ]  3, c Raf, MAPK kinase 1 2 MEK1 2, MAPK, Elk 1 and Akt 1.  Ref: 12888923 Int J Oncol, 2003
- Addition of epidermal growth factors receptor  **EGFR**  antibody to cells exposed to media devoid of EGF and media supplemented with TGF alpha  [ **TGFA** ]  showed marked suppression of proliferation of target cells.  Ref: 12593448 Indian J Cancer< MedlineTA>Indian J Cancer, 2003
**Neutral relationship**- In contrast, the amount of TGF alpha  [ **TGFA** ]  bound to  **EGFR**  is found to be higher in the gastric mucosa of aged than in young rats.  Ref: 12772780 Dig Dis Sci, 2003
- Our simulations predict that when  **EGFR**  is activated with TGF alpha  [ **TGFA** ] , receptor activation is biased toward the cell surface whereas EGF produces a signaling bias toward the endosomal compartment.  Ref: 12885624 Biophys J, 2003
- The epidermal growth factor receptor  [ **EGFR** ]  is a transmembrane glycoprotein that mediates the cellular responses to epidermal growth factor EGF and transforming growth factor alpha  [ **TGFA** ]  TGF alpha  [ **TGFA** ] .  Ref: 12731890 Biochemistry, 2003
- METHODS The pCMV AS  **EGFR** , a recombinant vector expressing antisense  **EGFR**  under the control of human CMV promotor was constructed and transfected the transformant PC 7 AS TGF alpha  [ **TGFA** ]  cell line cells, which had been transfected by a recombinant retroviral vector expressing antisense TGF alpha  [ **TGFA** ] .  Ref: 12569632 Zhongguo Yi Xue Ke Xue Yuan Xue Bao, 1999
- The epidermal growth factor EGF receptor  **EGFR**  is one of four homologous transmembrane proteins that mediate the actions of a family of growth factors including EGF, transforming growth factor alpha  [ **TGFA** ] , and the neuregulins.  Ref: 12648464 Exp Cell Res, 2003
- The TGF alpha  [ **TGFA** ]  induction of AP 1 transcriptional activity in gastric mucosal cells from aged rats to could be totally abrogated by either PD153035, a specific inhibitor of  **EGFR**  tyrosine kinase, or PD98059, a specific inhibitor of MEKs, but not by Wortmannin, which inhibits PI 3 kinase.  Ref: 12672650 Am J Physiol Gastrointest Liver Physiol< MedlineTA>Am J Physiol Gastrointest Liver Physiol, 2003

**Non-interactive relationship** (e.g. studied together, co-existance, homology, etc.)

- RESULT The expression of TGF alpha  [ **TGFA** ] ,  **EGFR**  and PCNA were increased in the epithelium, gland cells and inflammatory cells of nasal polyps.  Ref: 12563935 Lin Chuang Er Bi Yan Hou Ke Za Zhi, 2000
- There was a close correlation between the intensities of TGF alpha  [ **TGFA** ] ,  **EGFR**  and PCNA.  Ref: 12563935 Lin Chuang Er Bi Yan Hou Ke Za Zhi, 2000
- Comparative study in the expression of p53,  **EGFR** , TGF alpha  [ **TGFA** ] , and cyclin D1 in verrucous carcinoma, verrucous hyperplasia, and squamous cell carcinoma of head and neck region.  Ref: 12607604 Appl Immunohistochem Mol Morphol, 2002
- TGF alpha  [ **TGFA** ]  and  **EGFR**  expression, as determined by RNAse protection assay, was maximal at P6 in normal animals, but remained low in hypothyroid animals, suggesting that thyroid hormone was responsible for their induction.  Ref: 12524172 Brain Res Dev Brain Res, 2003
- Correlation between the level of expression of all markers and the grade of this group of squamous lesions revealed statistically significant correlation coefficients for p53 and  **EGFR**  but not for TGF alpha  [ **TGFA** ]  and cyclin D1.  Ref: 12607604 Appl Immunohistochem Mol Morphol, 2002
- OBJECTIVE Investigate the effects of blocking the TGF alpha  [ **TGFA** ]   **EGFR**  autocrine loop on the growth of human pancreatic carcinoma.  Ref: 12569632 Zhongguo Yi Xue Ke Xue Yuan Xue Bao, 1999
- This work examined the importance of radiation induced and ligand induced  **EGFR**  ERK signaling for the regulation of DNA repair proteins XRCC1 and ERCC1 in prostate carcinoma cells, DU145 TP53 mut, displaying  **EGFR**   **TGFA**  dependent autocrine growth and high MAPK ERK1 2 activity, and LNCaP TP53 wt cells expressing low constitutive levels of ERK1 2 activity.  Ref: 12643788 Radiat Res, 2003
- So, this study confirms the development of human olfactory mucosa and shows epidermal growth factor receptor  [ **EGFR** ] , transforming growth factor alpha  [ **TGFA** ]  and nerve growth factor beta proteins are reliable markers for developing olfactory epithelium.  Ref: 12739843 Growth Dev Aging< MedlineTA>Growth Dev Aging, 2003
- Antisense oligonucleotides directed against mRNA encoding transforming growth factor alpha  [ **TGFA** ]  and the epidermal growth factor receptor  [ **EGFR** ]  have demonstrated in vitro and in vivo efficacy against prostate cancer tumor models.  Ref: 12665682 Med Oncol< MedlineTA>Med Oncol, 2003
- Here we show that when its proteolytic shedding is prevented, the transmembrane form of the transforming growth factor alpha  [ **TGFA** ]  proTGF alpha interacts with, but does not activate, the  **EGFR** .  Ref: 12606576 EMBO J, 2003
- The similarity of the wavy coat phenotype to those of  **Tgfa**  and  **Egfr**  mutations and the map positions of cub and mcub suggest candidate genes that interact in the EGF receptor signal transduction pathway.  Ref: 12573256 Genomics, 2003
- Consistent with the concept that matrix metalloproteinase MMP activity is requisite for TGF alpha  [ **TGFA** ]  membrane release and ligand function, bile acid transactivation of  **EGFR**  and cell growth was blocked by an MMP inhibitor.  Ref: 12606307 Am J Physiol Gastrointest Liver Physiol< MedlineTA>Am J Physiol Gastrointest Liver Physiol, 2003
- Immunohistochemical procedures were performed to detect the following protein antigens CD99, interleukin IL 1alpha, IL 6, transforming growth factor TGF alpha  [ **TGFA** ] , conventional c protein kinase C cPKC alpha, cPKC betaII, phosphorylated p PKC alpha betaII, c kit CD117, platelet derived growth factor receptor PDGFR alpha, PDGFR beta, epidermal growth factor receptor  [ **EGFR** ]   **EGFR** , human epidermal growth factor receptor  [ **EGFR** ]  HER 2 neu, cathepsin D, angiotensin converting enzyme ACE, angiotensin II type 1 AT1 receptor, p21ras, the alpha subunit of farnesyl and geranylgeranyl transferase FTalpha GGTalpha, phospho p c Jun N terminal kinase p JNK, p p38 mitogen activated protein kinase MAPK, cyclin D1, c Jun, Ki 67, bcl 2, TGF beta1 latency associated peptide LAP, TGF betaRII, and cyclooxygenase COX 2.  Ref: 12817616 Ann Clin Lab Sci< MedlineTA>Ann Clin Lab Sci, 2003
- Although cells constitutively expressed several  **EGFR**  ligands, only transforming growth factor alpha  [ **TGFA** ]  TGF alpha  [ **TGFA** ]  antisera effectively blocked bile acid induced  **EGFR**  phosphorylation.  Ref: 12606307 Am J Physiol Gastrointest Liver Physiol< MedlineTA>Am J Physiol Gastrointest Liver Physiol, 2003
- TACE is required for the activation of the  **EGFR**  by TGF alpha  [ **TGFA** ]  in tumors.  Ref: 12606576 EMBO J, 2003
- Antisense oligonucleotides directed against mRNA encoding, transforming growth factor alpha  [ **TGFA** ]  and the epidermal growth factor receptor  [ **EGFR** ] , have been shown to significantly inhibit in vitro and in vivo growth of prostate tumor models.  Ref: 12616956 Methods Find Exp Clin Pharmacol, 2002
- CONCLUSION The expression levels of SF HGF and TGF alpha  [ **TGFA** ]  as well as their respective receptors, MET and  **EGFR** , are known to correlate with glioma malignancy grade.  Ref: 12762884 Neurosurgery, 2003
- Transforming growth factor alpha  [ **TGFA** ]  TGF alpha  [ **TGFA** ] , a ligand of the epidermal growth factor receptor  [ **EGFR** ] , reduces the infarct size after focal cerebral ischemia in rat but the molecular basis underlying the protection are unknown.  Ref: 12771152 J Biol Chem< MedlineTA>J Biol Chem, 2003
- The primary objective of this investigation is to determine the role of signaling pathways initiated by  **EGFR**  in regulating the TGF alpha  [ **TGFA** ]  induced activation of AP 1 in the gastric mucosa in aged rats.  Ref: 12672650 Am J Physiol Gastrointest Liver Physiol< MedlineTA>Am J Physiol Gastrointest Liver Physiol, 2003
- Effects of blocking the TGF alpha  [ **TGFA** ]   **EGFR**  autocrine loop on the growth of human pancreatic carcinoma cells in vitro < ArticleTitle>.  Ref: 12569632 Zhongguo Yi Xue Ke Xue Yuan Xue Bao, 1999
- Moreover, autocrine TGF alpha  [ **TGFA** ]  mediated epidermal growth factor receptor  [ **EGFR** ]  activation was shown to control the constitutive activation of the ERK MAPK pathway, since neutralizing antibody to the epidermal growth factor receptor  [ **EGFR** ]  was able to block basal ERK activity.  Ref: 12657625 J Biol Chem, 2003
- CONCLUSIONS These observations strongly support that the blockage of the expression of autocrine growth factor TGF alpha  [ **TGFA** ]  and its receptor  **EGFR**  was a potent way in circumventing the malignant properties of the pancreatic carcinoma cells.  Ref: 12569632 Zhongguo Yi Xue Ke Xue Yuan Xue Bao, 1999
- In this study, standard immunohistochemical methods were used to detect the presence of epidermal growth factor receptor  [ **EGFR** ] , transforming growth factor alpha  [ **TGFA** ]  and nerve growth factor beta in the human olfactory epithelium of different developmental stages 7 to 30 weeks in formalin fixed tissue specimens.  Ref: 12739843 Growth Dev Aging< MedlineTA>Growth Dev Aging, 1999
- Induction of DNA synthesis in primary mouse hepatocytes is associated with nuclear pro transforming growth factor alpha  [ **TGFA** ]  and erbb 1  [ **EGFR** ]  and is independent of c jun.  Ref: 12771026 Carcinogenesis, 2003
- Detection of transforming growth factor alpha  [ **TGFA** ]  and epidermal growth factor receptor  [ **EGFR** ]  mRNA and immunohistochemical localization of their proteins in the ovine uterus during the early implantation period.  Ref: 12814185 Histochem J< MedlineTA>Histochem J, 2003
- To determine the utility of this approach, we simulated the differential activation of the  **EGFR**  by two of its ligands, epidermal growth factor EGF and transforming growth factor alpha  [ **TGFA** ]  TGF alpha  [ **TGFA** ] .  Ref: 12885624 Biophys J, 2003
- Here we evaluated  **EGFR**  activation, Cbl recruitment,  **EGFR**  ubiquitination and degradation in response to EGF,  **TGFa**  and E4T.  Ref: 12829707 J Biol Chem< MedlineTA>J Biol Chem, 2003
- We hypothesize that the expression of epidermal growth factor receptor  [ **EGFR** ]   **EGFR** , epidermal growth factor EGF, and transforming growth factor alpha  [ **TGFA** ]  TGFalpha in GCT have a role in the development of PEH overlying some GCT.  Ref: 12715330 Ann Diagn Pathol, 2003
- Similarly,  **EGFR**  TK inhibition by ZD1839 reversed the invasive phenotype promoted by the constitutively activated form of the  **EGFR**  EGFRvIII and the  **EGFR**  agonists transforming growth factor alpha  [ **TGFA** ]  TGFalpha, amphiregulin and EGF.  Ref: 12881705 Oncogene, 2003
- In conclusion, bile acids activate  **EGFR**  via a TGF alpha  [ **TGFA** ]  dependent mechanism, and this  **EGFR**  activation promotes cellular growth.  Ref: 12606307 Am J Physiol Gastrointest Liver Physiol< MedlineTA>Am J Physiol Gastrointest Liver Physiol, 2003
- In this study, we investigated the effects of thyroid hormone on cerebellar development and the coincident expression of transforming growth factor alpha  [ **TGFA** ]  TGF alpha  [ **TGFA** ] , a ligand in the epidermal growth factor EGF family, and the epidermal growth factor receptor  [ **EGFR** ]   **EGFR** .  Ref: 12524172 Brain Res Dev Brain Res, 2003
- RT PCR transcripts of  **EGFR**  ligands EGF, TGF alpha  [ **TGFA** ] , HB EGF, and amphiregulin were detected in both carcinomas and normal mucosa, indicating that autocrine growth stimulation of colorectal carcinomas is mediated by coexpression of EGF receptor ligands and upregulation of EGF receptors.  Ref: 12790326 Recent Results Cancer Res, 2003
- G418 resistant colonies were isolated and identified as PC 7 AS TGF alpha  [ **TGFA** ]  AS  **EGFR** .  Ref: 12569632 Zhongguo Yi Xue Ke Xue Yuan Xue Bao, 1999
- OBJECTIVE To assess the possible role of expression of TGF alpha  [ **TGFA** ]  and  **EGFR**  in nasal polyps and its relationship with PCNA labeling index.  Ref: 12563935 Lin Chuang Er Bi Yan Hou Ke Za Zhi, 2000
- This study investigates the expression of p53, epidermal growth factor receptor  [ **EGFR** ]   **EGFR** , transforming growth factor alpha  [ **TGFA** ]  TGF alpha  [ **TGFA** ] , and cyclin D1 in VC, verrucous hyperplasia VH, and classic SCC of the head and neck.  Ref: 12607604 Appl Immunohistochem Mol Morphol, 2002
- Both cell lines were capable of generating a range of  **EGFR**  specific ligands and increased expression of transforming growth factor alpha  [ **TGFA** ]  was observed in TAM R cells.  Ref: 12586780 Endocrinology, 2003
- Using relatively low concentrations of oligos, we compared the efficacy of the first generation phosphorothioated oligos against TGF alpha  [ **TGFA** ]  and  **EGFR**  with second generation oligos containing completely phosphorothioated backbones and different patterns of 2 methoxyethyl backbone modifications, while retaining the original designated base sequence using, the LNCaP and PC 3 prostate cancer cell lines, respectively.  Ref: 12616956 Methods Find Exp Clin Pharmacol, 2002
- These effects appear to be due to the ability of TCDD to increase the expression of transforming growth factor alpha  [ **TGFA** ] , a known  **EGFR**  ligand.  Ref: 12668121 Toxicol Appl Pharmacol, 2003
- Bile Acids Activate Epidermal Growth Factor Receptor  [ **EGFR** ]  via a Transforming Growth Factor alpha  [ **TGFA** ]  dependent Mechanism in Human Cholangiocyte Cell Lines.  Ref: 12606307 Am J Physiol Gastrointest Liver Physiol< MedlineTA>Am J Physiol Gastrointest Liver Physiol, 2003
- Inhibition of ErbB  [ **EGFR** ]  2 and ErbB  [ **EGFR** ]  3 expression by quercetin prevents transforming growth factor alpha  [ **TGFA** ]  TGF alpha  [ **TGFA** ]  and epidermal growth factor EGF induced human PC 3 prostate cancer cell proliferation.  Ref: 12888923 Int J Oncol, 2003
- Taken together, these results implicate thyroid hormone in the coordinated expression of TGF alpha  [ **TGFA** ]  and  **EGFR** , which are positioned to play a role in post mitotic developmental events in the cerebellum.  Ref: 12524172 Brain Res Dev Brain Res, 2003
